# Change in glucose intolerance status and risk of incident cardiovascular disease: Tehran Lipid and Glucose Study

**DOI:** 10.1186/s12933-020-01017-4

**Published:** 2020-03-30

**Authors:** Maryam Kabootari, Mitra Hasheminia, Fereidoun Azizi, Mohammadhassan Mirbolouk, Farzad Hadaegh

**Affiliations:** 1 Metabolic Disorders Research Center, Golestan university of Medical Sciences, Gorgan, Iran; 2 Prevention of Metabolic Disorders Research Center, Research Institute for Endocrine Sciences, Shahid Beheshti University of Medical Sciences, No. 24, Yamen Street, Velenjak, P.O. Box: 19395-4763, Tehran, Iran; 3 Endocrine Research Center, Research Institute for Endocrine Sciences, Shahid Beheshti University of Medical Sciences, Tehran, Iran; 4 Internal Medicine Department, Yale School of Medicine, New Haven, USA

**Keywords:** Impaired fasting glucose, Impaired glucose tolerance, Cardiovascular disease

## Abstract

**Background:**

To assess the impact of changes in different glucose tolerance states on risk of incident cardiovascular disease (CVD)/coronary heart disease (CHD).

**Methods:**

A total of 4094 Iranians (43.9% men) aged ≥ 30 years, without diabetes and CVD at enrolment were included. The following categories were defined both at baseline visit and 3 years later (second visit): normal fasting glucose (NFG), normal glucose tolerance (NGT), NFG and NGT (NFG/NGT), impaired fasting glucose (IFG), impaired glucose tolerance (IGT) and IFG and/or IGT (IFG/IGT). Changes in the categories, i.e. regression to normoglycemia, remaining in previous status and progression to diabetes were assessed. We used Cox’s proportional hazard models adjusted for traditional risk factors and their changes, to estimate the hazard ratio (HR) with 95% confidence interval (CI) of different changing categories for incident CVD/CHD.

**Results:**

During a median follow-up of 12.42 years, 428 subjects (men = 265) experienced CVD. Considering persistent NFG/NGT as reference, participants who shifted from NFG/NGT to IFG/IGT showed a lower hazard of CVD in the fully adjusted model, HR 0.72 [95% CI 0.52–0.996, P = 0.048]. Moreover, subjects who shifted from IFG, IGT and IFG/IGT to diabetes had an increased risk of CVD/CHD. The risk however, was only statistically significant for those with IFG/IGT, 1.61 [(1.03–2.51), P = 0.04] for CVD and 1.75 [(1.10–2.78), P = 0.02] for CHD; considering IFG/IGT at both visits as reference. Furthermore, those who regressed from IFG/IGT to normoglycemia were at the same risk as those remained in IFG/IGT state, 1.12 [(0.79–1.60), P = 0.52] for CVD and 1.04 [(0.70–1.53), P = 0.85] for CHD. Among a subgroup of population with insulin data (n = 803) those with insulin resistance (IR) that converted to diabetes showed a higher risk for CVD, 3.68 [(1.49–9.06), P = 0.01] and CHD, 2.76 [(1.00–7.60), P = 0.05] events in the fully adjusted model.

**Conclusions:**

Among participants with IFG, IGT or IFG/IGT at baseline, only those who developed diabetes had a higher risk of developing CVD/CHD. Persistent IFG/IGT was not associated with higher risk, compared with those reverted to normoglycemia. Moreover, subjects who converted from NFG/NGT to incident IFG/IGT showed a signal for lower risk of CVD/CHD.

## Background

Prediabetes, defined as the presence of impaired fasting glucose (IFG), impaired glucose tolerance (IGT) or both, is considered a high-risk state for type 2 diabetes [1], hypertension [2], subclinical atherosclerosis [3] and cardiovascular disease (CVD) [4]. A national Iranian study [5] estimates that over 4 million Iranian adults had IFG in 2005. Prediabetes remains a public health priority for Iran given that another study highlighted that every year more than 4% of adult residents in Tehran with normal glucose metabolism develop prediabetes [6].

Currently, individuals with prediabetes are warned about the cardiovascular consequences of the condition and advised to initiate lifestyle modification [7]. Most of the previous studies have assessed the association between prediabetes and CVD were based on 1-time point measurement of blood glucose at the time of recruitment rather than assessing the change in blood glucose concentration over time [4]. The remaining question is that whether the reason behind this association is due to the direct effect of prediabetes or is mediated by conversion of prediabetes to diabetes state and whether regression from prediabetes to normoglycemia could decrease this risk. Some studies conducted among European, Korean and American populations have assessed this issue; some showed an increased risk of CVD in the presence of IFG and/or IGT per se (i.e. without changing to diabetes state) [8, 9], while others showed this risk was increased only after progression to type 2 diabetes [10, 11].

Previously during a 7-year follow-up, we found IFG/IGT was associated with 56% risk of CVD only in the age adjusted model in women [12]. Hence, considering the high prevalence and incidence of IFG [5, 13] as well as high CVD burden in the Middle East and North Africa (MENA) populations [14], we aimed to investigate whether remaining in the IFG, IGT and IFG/IGT (IFG and/or IGT) states, regression to normoglycemia, or conversion to diabetes state, during a 3-year period, is associated with the long term risk of CVD and coronary heart disease (CHD) in the oldest cohort of MENA, i.e. the Tehran Lipid and Glucose Study (TLGS).

## Methods

### Study population

The TLGS is a large longitudinal prospective population-based study of a representative urban sample of Tehran population. Details of study design, sampling and rationale is published elsewhere [15].

In brief, TLGS includes 15,005 participants at first visit (1999–2002), with additional 3550 recruitments in the second visit (2002–2005) of study. Follow up visits happened at approximately 3 year intervals. In the current study 9558 individuals, who were 30 years or older were included [7927 from baseline (1999–2002) and 1631 from second phase (2002–2005)]. After excluding those with diabetes (n = 1354), prevalent CVD (n = 406) or incident CVD before the second examination (n = 128), 7670 individuals remained. Other exclusion criteria included those who did not participate in the second examination i.e., 2002–2005 for those entered in the first phase and 2005–2008 for participants recruited in the second phase (n = 2810), those with missing data on covariates, i.e., age, sex, smoking, education, physical activity, creatinine, fasting plasma glucose (FPG), 2-h post challenge plasma glucose (2 h-PCPG), total cholesterol (TC), high density lipoprotein-cholesterol (HDL-C), body mass index (BMI), waist circumference (WC), systolic blood pressure (SBP) and diastolic blood pressure (DBP), at baseline and second examination (n = 766), leading to 4094 participants with complete data (respondents) who were followed until March 2016.

### Clinical and laboratory measurements

Demographic information, medical history, smoking habits and history of CVD were obtained from participants during interviews, using a pretested questionnaire at baseline and each follow-up. Details of anthropometric measurements including weight, height and WC have been previously described elsewhere [15]. BMI was calculated as weight in kilograms divided by square of height (m^2^). Blood pressure was measured using a standardized mercury sphygmomanometer (calibrated by the Iranian Institute of Standards and Industrial Researches), twice on the right arm in a seated position after at least 15 min rest and the mean of these two measurements, was considered as the participant’s blood pressure.

At the first phase of the TLGS, assessment of physical activity level was performed using the Lipid Research Clinics questionnaire; however, due to inexactness of this questionnaire, it was replaced by the Modifiable Activity Questionnaire in the second phase, which measures all 3 forms of activities including job, leisure time and household activities in the last year [15]. After 12 to 14 h of overnight fasting, a blood sample was taken for the biochemical analysis on the same day. FPG and 2 h-PCPG (using 75 g glucose, for those without history of taking glucose-lowering medications) were measured by the glucose oxidation enzymatic colorimetric method. Further details regarding laboratory measurements including FPG, 2-h PCPG, TC and HDL-C have been described before [15].

### Outcomes

As reported in our first article regarding CVD outcomes in the TLGS cohort [16], each participant is followed-up for any medical event leading to hospitalization during the past year by telephone call and he/she is asked for any medical conditions by a trained nurse. If a related event has occurred, a trained physician collects complementary data regarding that event during a home visit and by gathering data from participant’s medical files. In the case of mortality, data is collected from the hospital or death certificate by an authenticated local physician. Collected data is then evaluated by an outcome committee consisting of an internist, endocrinologist, cardiologist, epidemiologist, and other experts, when needed, to assign a specific outcome for every event. Importantly, the outcome committee is blinded to the status of baseline risk factors.

In the current study, CHD events included cases of (1) definite myocardial infarction (MI) diagnosed by diagnostic electrocardiogram (ECG) and biomarkers (including CK, CK-MB, CK-MBm, troponin (cTn) and myoglobin), (2) probable MI distinguished by positive ECG findings plus cardiac symptoms or signs and biomarkers showing negative or equivocal results, (3) unstable angina pectoris, who admitted the hospital and developed new cardiac symptoms or showed changing symptom patterns and positive ECG findings with normal biomarkers [17], (4) angiography proven CHD defined as ≥ 50% stenosis in at least one major coronary vessel [18], and (5) CHD death (any death due to CHD according to the above-mentioned criteria or sudden cardiac death caused by cardiac disease occurring ≤ 1 h after onset of symptoms according to verbal autopsy documents outside of hospital). Details of stroke definition in TLGS cohort has been addressed elsewhere [19]. Accordingly, definite stroke was defined using the World Health Organization’s definition as “rapidly developing clinical signs of focal or global disturbance of cerebral function, lasting > 24 h or leading to death with no apparent cause other than that of vascular origin” [20]. Moreover, another criterion of definite stroke was imaging suggestive of stroke in cases of acute clinically relevant brain injuries accompanied by rapidly vanishing symptoms. Possible stroke was defined as any acute neurologic deficit with no imaging that is indicative of stroke or with data that were not fully consistent with the World Health Organization’s definition for definite stroke. When symptoms resolved within 24 h, cases were labeled as transient ischemic attack. In the current study, all cases of definite or possible stroke or transient ischemic attack were defined as stroke. Furthermore, CVD was defined as a composite measure of any CHD events, stroke or cerebrovascular death.

### Definition of terms

We used the 2003 American Diabetes Association (ADA) criteria as our reference for categorization of our study. Therefore, we defined normal fasting glucose (NFG) as FPG < 5.6, normal glucose tolerance (NGT) as 2 h-PCPG < 7.8 mmol/L, IFG: 5.6 ≤ FPG < 7 mmol/L and IGT: 7.8 ≤ 2 h-PCPG < 11 mmol/L. NFG/NGT was defined as those with both NFG and NGT states. IFG/IGT was defined as those with IFG and/or IGT. Diabetes was defined as FPG ≥ 7 mmol/L, 2 h-PCPG ≥ 11.1 mmol/L or using anti-hyperglycemic agents.

Estimated glomerular filtration rate (eGFR) presented as mL/min/1.73 m^2^, was estimated using the CKD Epidemiology Collaboration (CKD-EPI) equation [21].

Smoking was defined as occasional or daily use of any kind of tobacco and smoking status was classified as current versus past or never smoker. Low physical activity was classified as subjects participating in physical activity < 3 day/week for participants recruited in first phase or < 600 metabolic equivalent task–minutes (MET)/week for those who entered in the second phase [22]. Education was classified into three groups: < 6 years, 6–12 years and > 12 years. Marital status was categorized as single, married and widowed/divorced.

### Statistical analysis

The baseline characteristics were presented as mean (standard deviation, SD) for continuous variables and frequencies (%) for categorical variables. Comparison of baseline characteristics between NFG/NGT versus IFG/IGT was done by Student’s T-test for continuous variables and the Chi-square test for categorical variables. The required assumptions to conduct the T-test are normal distribution of data and homogeneity of the variance. Normality was tested using Kolmogorov–Smirnov (K–S) test. Moreover, we also used histograms with fitted normal curves to check the normality of data. Homogeneity of variances was tested using the Levene test.

Mean difference [95% Confidence interval (CI)] of continuous variables and the mean differences in the prevalence [95% CI] of each category of categorical variables were estimated to compare respondents with non-respondents [those who did not participate in the first follow-up visit and those with missing data of FPG, 2 h-PCPG and other covariates (n = 3576)].

Cox proportional hazard regression was used to assess the hazard ratios (HRs) of changes in glucose tolerance status for CVD/CHD. Time to event was defined by time of censoring or the event occurring, whichever came first. We censored participants in the case of other causes of death, leaving the district or being in the study until 20 March 2016, without any event.

Univariable Cox analysis was performed for each potential risk factor including age, sex, education, using lipid lowering or anti-hypertensive drug, being in the intervention group, smoking, SBP, DBP, marital status, TC, HDL-C, BMI, WC, eGFR, physical activity as well as changes in BMI, WC, SBP, DBP, eGFR, TC and HDL-C; then, covariates with a P-value < 0.2 in the initial univariable analysis were selected to enter the multivariable model.

The following categories both at baseline and 3 years later were defined: NFG, NGT, NFG/NGT, IFG, IGT and IFG/IGT as well as incident type 2 diabetes (only in the first follow-up). We checked for changes for each different category over the next examination.

**Fig. 1:**
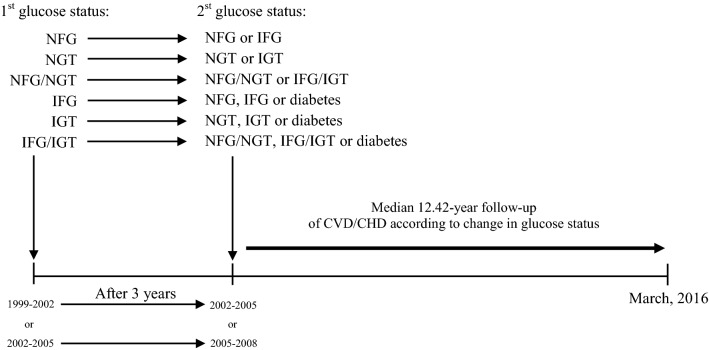
Timeline of the study design. Subjects without diabetes and cardiovascular disease at first visit (1999–2002) or (2002–2005) were followed 3 years later for the following changes: (1) Normal fasting glucose (NFG) to NFG or impaired fasting glucose (IFG), (2) Normal glucose tolerance (NGT) to NGT or impaired glucose tolerance (IGT), (3) NFG and NGT (NFG/NGT) to NFG/NGT or IFG/IGT, (4) IFG to NFG, IFG or diabetes, (5) IGT to NGT, IGT or diabetes and 6) IFG and/or IGT (IFG/IGT) to NFG/NGT, IFG/IGT or diabetes. Regarding the limited number of those with normoglycemia at baseline who directly converted to diabetes [NFG to diabetes (n = 60), NGT to diabetes (n = 52), NFG/NGT to diabetes (n = 24)], these groups were excluded

For those with NFG, NGT and NFG/NGT at baseline, changes include remaining in normoglycemia or progression to IFG, IGT or IFG/IGT. For those with IFG, IGT and IFG/IGT, regression to normoglycemia, remaining in previous status and progression to diabetes, were assessed (Fig. 1). Considering the limited number of those with NFG, NGT and NFG/NGT at baseline who directly converted to diabetes [NFG to diabetes (n = 60), NGT to diabetes (n = 52), NFG/NGT to diabetes (n = 24)], we excluded these groups in the data analysis.

Three models were defined: model 1 was adjusted for age, sex and model 2 was further adjusted for SBP, DBP, TC, HDL-C, WC, eGFR, physical activity, smoking, education and use of anti-hypertensive drug. In model 3, covariates in model 2 plus change of WC, TC and HDL-C were adjusted.

The proportional hazards assumption in the Cox model was assessed using Schoenfild residual test and all proportionality assumptions were appropriate. Statistical analysis was performed using SPSS for windows version 20 and STATA version 14. P-values ≤ 0.05 were considered statistically significant.

## Results

Baseline characteristics are presented in Table 1. The mean (SD) age of the total population was 45.5 (11.4) years and 43.9% were men. Participants in the NFG/NGT group were younger with more favorable metabolic profile and higher level of education than those who were in the IFG/IGT group. However, participants with IFG/IGT at baseline had more favorable change in anthropometric measures, DBP, TC, FPG and 2 h-PCPG between first and second examination compared to NFG/NGT group. Furthermore, as shown in Additional file 1: Table S1, baseline characteristics of respondent and non-respondent groups were similar.

**Table 1:** Baseline characteristics of study participants: Tehran Lipid and Glucose Study

	NFG/NGT (n = 3027)	IFG/IGT (n = 1067)	Total population (n = 4094)	P value
Continuous variables				
Age (years)	44.2 (11.1)	49.3 (11.6)	45.5 (11.5)	< 0.001
SBP (mmHg)	116.6 (16.9)	126.0 (19.3)	119.1 (18.1)	< 0.001
SBP change (mmHg)	− 1.19 (13.1)	− 2.07 (15.5)	− 1.42 (13.83)	0.07
DBP (mmHg)	76.8 (10.3)	81.2 (10.8)	77.9 (10.6)	< 0.001
DBP change (mmHg)	− 1.9 (9.09)	− 3.2 (9.5)	− 2.3 (9.20)	< 0.001
BMI (kg/m^2^)	27.0 (4.3)	28.6 (4.4)	27.4 (4.3)	< 0.001
BMI change (kg/m^2^)	0.8 (1.9)	0.5 (1.9)	0.74 (1.90)	< 0.001
WC (mmol/L)	88.5 (10.7)	93.8 (10.8)	89.9 (11.0)	< 0.001
WC change (mmol/L)	3.9 (6.6)	3.16 (6.18)	3.70 (6.54)	0.002
TC (mmol/L)	5.4 (1.1)	5.7 (1.2)	5.5 (1.1)	< 0.001
TC change (mmol/L)	− 0.30 (0.76)	− 0.45 (0.84)	− 0.34 (0.78)	< 0.001
HDL-C (mmol/L)	1.08 (0.28)	1.08 (0.29)	1.08 (0.28)	0.78
HDL-C change (mmol/L)	− 0.07 (0.23)	− 0.08 (0.25)	− 0.07 (0.24)	0.20
FPG (mmol/L)	4.8 (0.4)	5.6 (0.5)	5.03 (0.5)	< 0.001
FPG change (mmol/L)	0.1 (0.5)	− 0.001 (0.8)	0.08 (0.61)	< 0.001
2 h-PCPG (mmol/L)	5.5 (1.1)	7.6 (1.7)	6.07 (1.6)	< 0.001
2 h-PCPG change (mmol/L)	0.3 (1.6)	0.02 (2.6)	0.22 (1.92)	< 0.001
eGFR (mL/min/1.73 m^2^)	70.6 (11.0)	67.5 (10.8)	69.8 (11.0)	< 0.001
eGFR change (mL/min/1.73 m^2^)	2.9 (10.6)	4.4 (11.0)	3.35 (10.72)	< 0.001
Categorical variables				
Gender (males)	1334 (44.1%)	463 (43.4%)	1797 (43.9%)	0.72
Current smoker	421 (13.9%)	107 (10.0%)	528 (12.9%)	0.001
Hypertension	493 (16.3%)	337 (33.5%)	850 (20.8%)	< 0.001
Hypercholesterolemia	1659 (54.8%)	723 (67.8%)	2382 (58.2%)	< 0.001
Antihypertensive drug	130 (4.3%)	107 (10.0%)	237 (5.8%)	< 0.001
Lipid-lowering drug	58 (1.9%)	47 (4.4%)	105 (2.6%)	< 0.001
Low physical activity^a^	2040 (67.4%)	761 (71.3%)	2801 (68.4%)	0.01
Education				< 0.001
< 6 years	408 (13.5%)	107 (10.0%)	515 (12.6%)	
6–12 years	1600 (52.9%)	438 (41.0%)	2038 (49.8%)	
> 12 years	1019 (33.7%)	522 (48.9%)	1541 (37.6%)	
Marital status				0.002
Single	134 (4.4%)	21 (2%)	155 (3.8%)	
Married	2733 (90.3%)	979 (91.8%)	3712 (90.7%)	
Divorced/widowed	160 (5.3%)	67 (6.3%)	227 (5.5%)	

Values are expressed as mean (SD) for continuous variables and n (%) for categorical variables

NFG, normal fasting glucose; NGT, normal glucose tolerance; SBP, systolic blood pressure; DBP, diastolic blood pressure; BMI, body mass index; WC, waist circumference; TC, total cholesterol; HDL-C, high density lipoprotein-cholesterol; FPG, fasting plasma glucose; 2 h-PCPG, 2-h post challenge plasma glucose; eGFR, estimated glomerular filtration rate. NFG/NGT defined as those with both NFG and NGT states. IFG/IGT was defined as those with IFG and/or IGT

^a^Low physical activity was described as subjects participating in physical activity < 3 day/week for participants recruited in first phase or < 600 metabolic equivalent task–minutes (MET)/week for those who entered in the second phase

Of the total of 4094 participants without diabetes and CVD at baseline over a median follow up of 12.4 (interquartile range: 10.9–13.5) years (after second examination), 428 CVD and 368 CHD occurred. The corresponding incidence rates were 72.87 (66.29–80.11) and 62.39 (56.33–69.11) per 10,000 persons-years, respectively.

**Fig. 2:**
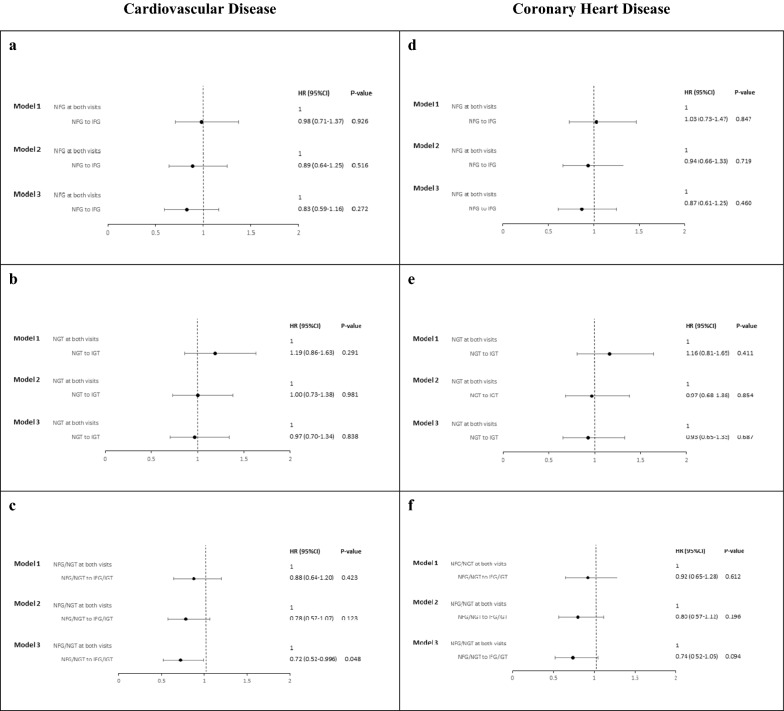
HRs (95% CI) of CVD and CHD for subjects with incident IFG, IGT and IFG/IGT. HR, hazard ratios; CI, confidence interval; CVD, cardiovascular disease; CHD, coronary heart disease; NFG, normal fasting glucose; IFG, impaired fasting glucose; NGT, normal glucose tolerance; IGT, impaired glucose tolerance. NFG/NGT was defined as those with both NFG and NGT states. IFG/IGT was defined as those with IFG and/or IGT. Model 1: Age, sex. Model 2: Model 1 + systolic blood pressure, diastolic blood pressure, total cholesterol, high density lipoprotein-cholesterol, waist circumference, eGFR, physical activity, smoking, education and use of anti-hypertensive drugs. Model 3: Model 2 + change of waist circumference, total cholesterol and high density lipoprotein-cholesterol. **a** Hazard of CVD in subjects with incident IFG compared to those with NFG in both visits; **b** hazard of CVD in subjects with incident IGT compared to those with NGT in both visits; **c** hazard of CVD in subjects with incident IFG/IGT compared to those with NFG/NGT in both visits; **d** hazard of CHD in subjects with incident IFG compared to those with NFG in both visits; **e** hazard of CHD in subjects with incident IGT compared to those with NGT in both visits; **f** hazard of CHD in subjects with incident IFG/IGT compared to those with NFG/NGT in both visits

As shown in Fig. 2, incident IFG, IGT and IFG/IGT cases had no higher hazards for developing CVD or CHD compared to the reference group i.e., normal glucose status in both baseline and first follow-up, in different models, in fact, among the NFG/NGT groups who converted to prediabetes, we found 28% lower risk of CVD events, HR, 0.72 [95% CI 0.52–0.996, P = 0.048].

**Fig. 3:**
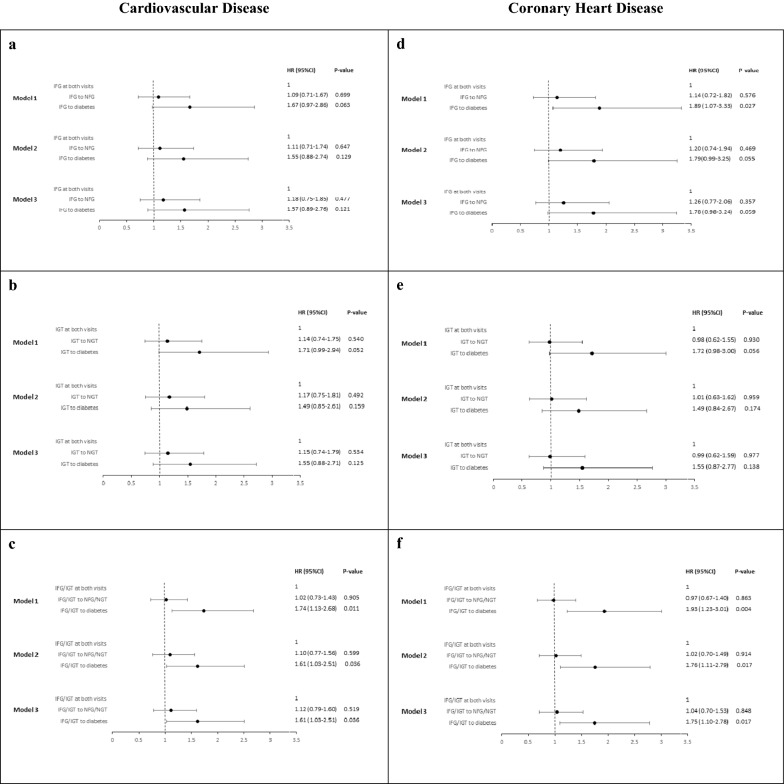
HRs (95% CI) of CVD and CHD for subjects with glucose intolerance at the first visit with their changing status at first follow-up. HR, hazard ratios; CI, confidence interval; CVD, cardiovascular disease; CHD, coronary heart disease; NFG normal fasting glucose; IFG, impaired fasting glucose; NGT, normal glucose tolerance; IGT, impaired glucose tolerance. NFG/NGT was defined as those with both NFG and NGT states. IFG/IGT was defined as those with IFG and/or IGT. Model 1: Age, sex. Model 2: Model 1 systolic blood pressure, diastolic blood pressure, total cholesterol, high density lipoprotein-cholesterol, waist circumference, eGFR physical activity, smoking, education and use of anti-hypertensive drug. Model 3: Model 2 + change of waist circumference, total cholesterol and high density lipoprotein-cholesterol. **a** Hazard of CVD in subjects with IFG at the first examination; **b** hazard of CVD in subjects with IGT at the first examination; **c** hazard of CVD in subjects with IFG/IGT at the first examination; **d** hazard of CHD in subjects with IFG at the first examination; **e** hazard of CHD in subjects with IGT at the first examination; **f** hazard of CHD in subjects with IFG/IGT at the first examination

Figure 3 summaries the hazard ratios of CVD/CHD for participants with IFG, IGT and IFG/IGT at baseline with changing status to normoglycemia or diabetes at the first follow-up. Participants who converted from IFG to NFG had similar risk of CVD as those with sustained IFG, while the HRs (95% CI) for those who progressed to diabetes were 1.67 [(0.97–2.86), P = 0.063] in model 1, 1.55 [(0.88–2.74), P = 0.129] in model 2 and 1.57 [(0.89–2.76), P = 0.121] in model 3. As for incident CHD, these values were 1.89 [(1.07–3.33), P = 0.027], 1.79 [(0.99–3.25), P = 0.055] and 1.78 [(0.98–3.24), P = 0.059] respectively.

Considering IGT group, for CVD/CHD events those who regressed to NGT showed similar risk as the reference group and a higher hazard upon conversion to diabetes with the HR (95% CI) of 1.71 [(0.99–2.94), P = 0.052] in model 1, 1.49 [(0.85–2.61), P = 0.159] in model 2 and 1.55 [(0.88–2.71), P = 0.125] in model 3 for developing CVD; similar associations were seen for incident CHD as the outcome.

Focusing on IFG/IGT, compared with persistent IFG/IGT at both visits, HRs (95% CI) of 1.74 (1.13–2.68), 1.61 (1.03–2.51), and 1.61 (1.03–2.51), for CVD and 1.93 (1.23–3.01), 1.76 (1.11–2.79), and 1.75 (1.10–2.78), for CHD in models 1, 2 and 3, respectively, were observed in those who converted from IFG/IGT to diabetes. (All P-values < 0.05). As in the IFG and IGT groups, the association between regression from IFG/IGT to NFG/NGT was similar to those of the reference group.

To show robustness of our findings, we repeated our data analysis among 803 subjects with available insulin data at baseline visit and first follow-up (Table 2). Insulin resistance (IR) was measured by the hemostasis model assessment-insulin resistance (HOMA-IR) index. Accordingly, IR and insulin sensitive (IS) among men was defined as HOMA-IR ≥ 2.17 µU/mL and < 2.17 µU/mL, respectively; corresponding values form women were ≥ 1.85 µU/mL and < 1.85 µU/mL respectively [23]. Results were generally in line with those of the main findings among all population and showed that those who converted from IR to diabetes were at greater risk of CVD 3.68 [(1.49–9.06), P = 0.01] and CHD, 2.76 [(1.00–7.60), P = 0.05] events in the fully adjusted model. However, this effect size was statistically unstable; considering wide CI due to limited number of events in the IR group that converted to diabetes (n = 25).

**Table 2:** HRs (95% (CI) of CVD and CHD for subjects with incident IR and those with changing IR status between baseline and the first follow-up (n = 803)

	Model 1^a^		Model 2^b^		Model 3^c^	
	HR 95% CI	P-value	HR 95% CI	P-value	HR 95% CI	P-value
Incident IR						
CVD						
IS in both visits	1.00		1.00		1.00	
IS to IR	1.02 (0.66–1.60)	0.91	0.85 (0.54–1.36)	0.50	0.80 (0.50–1.28)	0.35
CHD						
IS in both visits	1.00		1.00		1.00	
IS to IR	1.08 (0.67–1.73)	0.76	0.93 (0.57–1.53)	0.79	0.90 (0.54–1.49)	0.67
Changing IR						
CVD						
IR in both visits	1.00		1.00		1.00	
IR to IS	1.07 (0.62–1.85)	0.80	1.25 (0.72–2.19)	0.42	1.40 (0.79–2.48)	0.25
IR to diabetes	3.30 (1.40–7.80)	0.01	3.74 (1.55–9.03)	0.01	3.68 (1.49–9.06)	0.01
CHD						
IR in both visits	1.00		1.00		1.00	
IR to IS	1.23 (0.69–2.18)	0.48	1.47 (0.82–2.64)	0.20	1.53 (0.84–2.78)	0.17
IR to diabetes	2.73 (1.06–7.02)	0.04	3.21 (1.21–8.53)	0.01	2.76 (1.00–7.60)	0.05

Incident IR, those who converted from IS state to IR state. IR and IS among men was defined as HOMA-IR ≥ 2.17 µU/mL and < 2.17 µU/mL, respectively; corresponding values form women were ≥ 1.85 µU/mL and < 1.85 µU/mL respectively [23]

IR, insulin resistance; IS, insulin sensitive; HR, hazard ratios; CI, confidence interval; CVD, cardiovascular disease; CHD, coronary heart disease

^a^Model 1: Age, sex

^b^Model 2: Model 1 + systolic blood pressure, diastolic blood pressure, total cholesterol, high density lipoprotein-cholesterol, waist circumference, eGFR, physical activity, smoking, education and use of anti-hypertensive drugs

^c^Model 3: Model 2 + change of waist circumference, total cholesterol and high density lipoprotein-cholesterol

## Discussion

In the present study, we found that newly developed prediabetes (regardless of its definition) showed no higher risk for developing CVD/CHD. Moreover, only those with IFG/IGT who progressed to diabetes were at 61% and 75% significant higher risk of CVD and CHD, respectively, after adjustment for important traditional CVD risk factors along with their changes. Finally, regression from prediabetes with any definition to normal glucose state was associated with the same risk of incident CVD/CHD as persistent prediabetes state.

IFG and IGT have different underlying pathophysiological mechanisms and IGT is associated with more skeletal muscle (peripheral) insulin resistance than IFG. While IFG is characterized by hepatic insulin resistance and excessive endogenous glucose production [24, 25]. However, the impact of IFG and IGT on CVD are almost similar. For example, in a meta-analysis conducted by Huang et al., among 53 prospective cohorts [4] the association between baseline prediabetes state and risk of future CVD was assessed, and showed that prediabetes with different definitions [IFG-ADA (relative risk 1.13, 95% CI 1.05 to 1.21), IFG-WHO (1.26, 1.12 to 1.41) and impaired glucose tolerance (1.30, 1.19 to 1.42)], was associated with a relatively similar risk for composite cardiovascular disease. Nevertheless, the results were on the basis of a “snapshot” measurement of blood glucose, and the authors did not consider isolated IFG and IGT groups. A few studies with varying estimates of the CVD risk have assessed the impact of IFG/IGT with or without conversion to diabetes [8–11]; however, it’s difficult to compare the results due to the different sample size, age of participants, duration of changes in glucose category states and type and number of confounders [26].

Our results regarding the association between incident IFG and risk of CVD, were in line with those of a Korean population [11] showing no higher hazard for CVD. Moreover, in our study we found an unexpected finding regarding those with NFG/NGT who converted to IFG/IGT that showed 28% lower risk of CVD. As shown in Table 1, those with prevalent IFG/IGT at baseline had more favorable change in anthropometric measures, DBP as well as TC, FPG and 2 h-PCPG between first and second visit when compared to NFG/NGT group. We speculate that this favorable trend continued for those with newly developed IFG/IGT after baseline recruitment due to more knowledge of participants for controlling CVD risk factors after being diagnosed as prediabetics. In line with our findings, Diabetes Prevention Program Outcome Study (DPPOS) [27], showed that although a 18% per 10-year estimated CVD risk was seen among those individuals with persistent prediabetes, the trajectory of the estimated 10-year CVD risk decreased; this issue was mainly related to better control of TC and LDL-C (even compared with those who converted to normoglycemia) due to use of lipid lowering medications.

In the current study, considering different prediabetes definitions regression to normoglycemia was not associated with lower risk of CVD. This is in line with a study conducted among Korean population [11] showing that conversion from IFG to NFG, was not associated with a more favorable outcome; however, in the DPPOS study [27], regression from prediabetes to normoglycemia by receiving different interventions (i.e., metformin or lifestyle change) reduced risk of CVD. Moreover, researchers of the Whitehall II cohort study [28] recently showed that, only individuals with IGT (not those with prediabetes defined by HBA1c or FPG levels) who reverted to normoglycaemia, experienced a significant reduction in CVD risk.

Regarding persistent prediabetes status or conversion to diabetes, findings of our study supported the findings of previous studies including the study conducted among Korean population [11] which demonstrated that only conversion from IFG to diabetes was associated with an increased risk of myocardial infarction (MI), 1.65 [(1.20–2.27)], compared to persistent IFG state. Moreover, our data analysis showed a marginally significant higher risk of CHD for those with IFG who progressed to diabetes in the multivariate model. Furthermore, among a Dutch population [10], only subjects who converted from IFG to diabetes were at higher risk for CVD mortality in the age and sex adjusted analysis. However, subjects with persistent IFG had no higher risk than NFG subjects. Findings of a case–control study of the Framingham Heart Study collected on the offspring cohort participants [29], demonstrated that early onset (vs. late onset) IFG without progression to diabetes was associated with higher odds of CHD death compared to persistent NFG. Unfortunately, since we did not have the power to stratify our participants according to the prediabetes’ age of onset, it was hard to compare our findings with those of this study considering the effect of prediabetes’ age of onset on the risk of CVD.

In addition, in the Finnish study [8], subjects with IGT who did not develop diabetes after 10 years of follow-up had a non-significant 49% higher risk for CHD events, a risk which reached significant levels only for those who developed diabetes in the follow-up period. Similarly, in our study population, those with at least 3 years of persistent IGT showed no difference in CHD risk compared to those who converted to NGT. Moreover, among those with IR who converted to diabetes we found a significant higher risk of CVD/CHD; despite the insufficient number of events in these groups.

The strengths of our study include its prospective, longitudinal design with over a decade follow-up. Moreover, careful adjustment was performed for potential confounders and their changes over time. Finally, this is the first population-based cohort study from the MENA region with high burden of CVD events [30] which examined the impact of changing in different glucose tolerance status on risk of incident CVD/CHD events. There are of course, limitations to our study that should be noted: firstly, we did not have information to reliably estimate the onset of glucose intolerance at the baseline examination. Secondly, we had limited number of events to stratify our results according to sex. Thirdly, we did not have data of HbA1c which may lead to misclassification and underestimation of the CVD risk associated with prediabetes. Fourthly, we did not have enough events for stratifying prediabetes groups as isolated ones for example, isolated IFG. Fifthly, the data of ECG was not available for all of the participants at the baseline recruitment as well as during follow-up. Hence, silent CHD was not considered as part of either the exclusion criteria or CHD outcome. Sixthly, insulin data was not available for all participants; despite this, we studied a subgroup of our population with measurements of insulin level and showed a signal for higher risk of CVD/CHD among those who converted from IR to diabetes. Finally, this study was conducted on an Iranian population and the findings cannot be extrapolated to other ethnicities.

## Conclusion

The present study, during about 12-year follow-up, showed that the association between prediabetes and CVD is only present after progression to diabetes; moreover, regression to normoglycemia has no significant impact on development of CVD/CHD. Nevertheless, conversion from NFG/NGT to incident IFG/IGT showed a signal for lower risk of CVD.

## Supplementary information

**Additional file 1.** Comparison of baseline characteristics between respondent and non-respondent groups: Tehran Lipid and Glucose Study.

## Data Availability

All data and materials are available upon request.

## Abbreviations

CVD: Cardiovascular disease
CHD: Coronary heart disease
NFG: Normal fasting glucose
NGT: Normal glucose tolerance
NFG/NGT: NFG and NGT
IFG: Impaired fasting glucose
IGT: Impaired glucose tolerance
HR: Hazard ratio
CI: Confidence interval
MENA: Middle East and North Africa
TLGS: Tehran Lipid and Glucose Study
FPG: Fasting plasma glucose
2 h-PCPG: 2-h post challenge plasma glucose
BMI: Body mass index
WC: Waist circumference
SBP: Systolic blood pressure
DBP: Diastolic blood pressure
TC: Total cholesterol
HDL-C: High density lipoprotein-cholesterol
ECG: Electrocardiogram
ADA: American Diabetes Association
eGFR: Estimated glomerular filtration rate
CKD-EPI: CKD Epidemiology Collaboration
MET: Metabolic equivalent task—minutes
SD: Standard deviation
DPPOS: Diabetes Prevention Program Outcome Study
MI: Myocardial infarction
