# Supplementary material for: Change in glucose intolerance status and risk of incident cardiovascular disease: Tehran Lipid and Glucose Study
Source: Cardiovasc Diabetol. 2020 Mar 30;19:41. doi: 10.1186/s12933-020-01017-4 (PMC7106714; doi:10.1186/s12933-020-01017-4)
Supplement: Supplementary file 1 — Additional file 1. Comparison of baseline characteristics between respondent and non-respondent groups: Tehran Lipid and Glucose Study. [file 12933_2020_1017_MOESM1_ESM.docx]

| **Table S1. Comparison of baseline characteristics between respondent and non-respondent groups: Tehran Lipid and Glucose Study.** | | | |
| --- | --- | --- | --- |
| Mean (SD) or n (%) | Respondents  n=4094 | Non-respondents  n=3576 | Mean/Proportion difference (95% CI) |
| **Continuous variables** |  |  |  |
| Age (years) | 45.54(11.48) | 45.17(12.54) | 0.37(-0.17 to 0.90) |
| SBP (mmHg) | 119.08(18.1) | 118.97(18.5) | 0.79(-0.72 to 0.95) |
| DBP (mmHg) | 77.9 (10.6) | 77.8 (10.9) | 0.67(-0.38 to 0.59) |
| BMI (kg/m^2^) | 27.4 (4.4) | 27.1 (4.7) | 0.26 (0.05 to 0.47) |
| WC (cm) | 89.9 (11.0) | 89.4 (11.7) | 0.47(-0.05 to 0.99) |
| TC (mmol/L) | 5.48 (1.14) | 5.40 (1.14) | 0.08(0.03 to 0.13) |
| HDL-C (mmol/L) | 1.08(0.28) | 1.08(0.29) | 0.001(-0.01 to 0.01) |
| FPG (mmol/L) | 5.03 (0.5) | 5.04 (0.5) | -0.003(-0.03 to 0.02) |
| 2h-PCPG (mmol/L) | 6.07 (1.62) | 6.07 (1.68) | 0.0007(-0.08 to 0.08) |
| eGFR (mL/min/1.73m^2^) | 69.8(11.05) | 70.2(11.7) | -0.42(-0.94 to 0.10) |
| **Categorical variables** |  |  |  |
| Gender (males) | 1797(43.9%) | 1662(46.5%) | -0.02(-0.05 to -0.003) |
| Current smoker | 528(12.9%) | 693(20.2%) | -0.07 (-0.09 to -0.06) |
| Hypertension | 850 (20.8%) | 713 (21.0%) | -0.002(-0.02 to 0.02) |
| Hypercholesterolemia | 2382 (58.2%) | 1854 (55.8%) | 0.02(0.0007 to 0.05) |
| Anti-hypertensive drug use | 237(5.8%) | 205(5.7%) | 0.0005(-0.009 to 0.01) |
| Lipid-lowering drug | 105(2.6%) | 88(2.5%) | 0.001(-0.006 to 0.008) |
| Low physical activity | 2801(68.4%) | 2417(71.1%) | -0.03(-0.05 to -0.006) |
| Education |  |  |  |
| <6 | 1541(37.6%) | 1303(36.6%) | 0.01(-0.01 to 0.03) |
| 6-12 | 2038 (49.8%) | 1758(49.4%) | 0.004(-0.02 to 0.03) |
| >12 | 515(12.6%) | 501(14.1%) | -0.01(-0.03 to 0.00) |
| Marital status |  |  |  |
| Single | 155(3.8%) | 199(5.6%) | -0.02(-0.03 to -0.008) |
| Married | 3712 (90.7%) | 3125(87.4%) | 0.03(0.02 to 0.05) |
| Divorced/ Widowed | 227(5.5%) | 251(7.0%) | -0.015(-0.03 to -0.004) |
| Values are expressed as mean (SD) for continuous variables and n (%) for categorical variables.  SBP, systolic blood pressure; DBP, diastolic blood pressure; BMI, body mass index; WC, waist circumference; TC, total cholesterol; HDL-C, high density lipoprotein-cholesterol; FPG, fasting plasma glucose; 2h-PCPG, 2-h post challenge plasma glucose; eGFR, estimated glomerular filtration rate. | | | |
